# RNAi-directed downregulation of *OsBADH2 *results in aroma (2-acetyl-1-pyrroline) production in rice (*Oryza sativa L*.)

**DOI:** 10.1186/1471-2229-8-100

**Published:** 2008-10-08

**Authors:** Xiangli Niu, Wei Tang, Weizao Huang, Guangjun Ren, Qilin Wang, Di Luo, Yingyong Xiao, Shimei Yang, Feng Wang, Bao-Rong Lu, Fangyuan Gao, Tiegang Lu, Yongsheng Liu

**Affiliations:** 1 Ministry of Education Key Laboratory for Bio-resource and Eco-environment, College of Life Science, Sichuan University, Chengdu 610064, PR China; 2 State Key Laboratory of Hydraulics and Mountain River Engineering, Sichuan University, Chengdu 610064, PR China; 3 Institute of Crop Research, Sichuan Academy of Agricultural Sciences, Chengdu 610066, PR China; 4 Biotechnology Research Institute, Fujian Academy of Agricultural Sciences, Fuzhou 350003, PR China; 5 Ministry of Education Key Laboratory for Biodiversity Science and Ecological Engineering, Institute of Biodiversity Science, Fudan University, Shanghai 200433, PR China; 6 Biotechnology Research Institute, Chinese Academy of Agricultural Sciences, Beijing 100081, PR China

## Abstract

**Background:**

Aromatic rice is popular worldwide because of its characteristic fragrance. Genetic studies and physical fine mapping reveal that a candidate gene (*fgr*/*OsBADH2*) homologous to *betaine aldehyde dehydrogenase *is responsible for aroma metabolism in fragrant rice varieties, but the direct evidence demonstrating the functions of *OsBADH2 *is lacking. To elucidate the physiological roles of *OsBADH2*, sequencing approach and RNA interference (RNAi) technique were employed to analyze allelic variation and functions of *OsBADH2 *gene in aroma production. Semi-quantitative, real-time reverse transcription-polymerase chain reaction (RT-PCR), as well as gas chromatography-mass spectrometry (GC-MS) were conducted to determine the expression levels of *OsBADH2 *and the fragrant compound in wild type and transgenic *OsBADH2*-RNAi repression lines, respectively.

**Results:**

The results showed that multiple mutations identical to *fgr *allele occur in the 13 fragrant rice accessions across China; *OsBADH2 *is expressed constitutively, with less expression abundance in mature roots; the disrupted *OsBADH2 *by RNA interference leads to significantly increased 2-acetyl-1-pyrroline production.

**Conclusion:**

We have found that the altered expression levels of *OsBADH2 *gene influence aroma accumulation, and the prevalent aromatic allele probably has a single evolutionary origin.

## Background

Characteristic fragrance in aromatic rice leads to its popularity worldwide [1,2] and the aromatic traits have been extensively incorporated into commercial and hybrid rice breeding programs [3-5]. The fragrance occurring in many aromatic rice varieties has been shown to be associated with the presence of 2-acetyl-1-pyrroline [6], which has also been identified in a great variety of food products [7]. In the Thailand fragrant rice variety Khao Dawk Mali 105, 2-acetyl-1-pyrroline formation and strong aroma emission has been found to be positively correlated with an accumulation of proline [8]. Although the biochemical pathway leading to the fragrance is largely unknown, L-proline has been demonstrated to be a possible precursor in the production of 2-acetyl-1-pyrroline in rice plants [9].

The inheritance of fragrant trait in rice has been well documented. Genetic investigations implicated that the fragrant trait of rice was controlled by a single recessive locus [10,11]. A number of genetic analyses using reciprocal crosses repetitively showed the aromatic trait is characteristics of recessive monogenic inheritance without impact from cytoplasmic genes [12-14]. Nevertheless, Tsuzuki & Shimokawa [15] reported that two genes were responsible for the construction of the aromatic trait. In addition, several investigations by using different aromatic rice cultivars also showed that two recessive genes were involved in the segregation of aromatic and non-aromatic traits [16,17]. Furthermore, genetic studies revealed that the underlying gene responsible for the aroma production was located on chromosome 8 [18,19]. By using translocation and trisomics lines derived from non-fragrant rice cv. IR36, aromatic trait was also mapped to the chromosome 8 [20]. In a recent study involved eight aromatic hybrid rice maintainer lines, a single recessive locus spanning SSR (simple sequence repeat) markers RM210 and RM515 on chromosome 8 was identified to tightly link with the fragrant trait [17]. Physical mapping revealed that several candidate genes including a rice *betaine aldehyde dehydrogenase *(*OsBADH2*) homolog on chromosome 8 was co-segregated with aroma production [21]. Meanwhile, a delicate study demonstrated that the aroma production in fragrant genotypes was well correlated with the multiple mutations in the *fragrance rice *(*fgr*) locus that is identical to *betaine aldehyde dehydrogenase *(*OsBADH2*) on chromosome 8 [22]. However, direct evidence proving the function of *OsBADH2 *in 2-acetyl-1-pyrroline production is lacking. Furthermore, traditional aromatic cultivars have often undesirable agronomic performance, such as poor yield, susceptibility to pests and diseases, and strong shedding [3,10]. The molecular mechanism of this major weakness occurred in the aromatic rice cultivars is largely unknown.

In present experiments, we sequenced the *OsBADH2 *locus derived from a number of aromatic rice cultivars across China and uncovered that multiple mutations identical to *fgr *allele also occurred in all the tested fragrant accessions. By using RNA interference (RNAi) technique combined with *Agrobacterium tumefaciens-*mediated T-DNA transfer, we show that the directed degradation of *OsBADH2 *transcripts results in a significantly elevated fragrance emission and 2-acetyl-1-pyrroline accumulation, unambiguously suggesting that *OsBADH2* locus is responsible for aroma production in fragrant rice varieties. In addition, disrupted *OsBADH2 *leads to a detectable reduction of crop productivity, implying its multiple functions in secondary metabolism and agronomic performance. A strategy to compromise the favorable aspects and to avoid the unfavorable effect during *OsBADH2 *manipulation and breeding is discussed as well.

## Results

### Expression and nucleotide variation at the OsBADH2 locus

**Figure 1:**
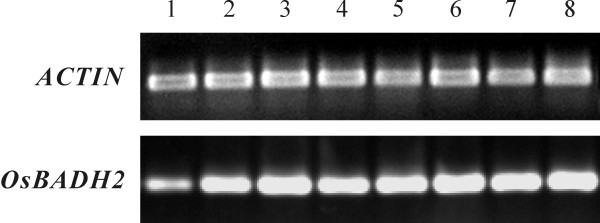
**Expression pattern of *OsBADH2 *in various tissues.** Expression abundance in root (lane 1), stem (lane 2), leaf (lane 3) and flower (lane 4) of mature rice plant, and embryo, seedling root, stem, leaf (lane 5, lane 6, lane 7, and lane 8, respectively) of cv. Nipponbare is shown, respectively. *ACTIN *was amplified as internal positive control.

The cv. Nipponbare was used to determine the expression patterns of *OsBADH2*. Total RNAs were extracted from roots, stems, leaves and flowers of mature plant, as well as embryos, seedling roots, seedling stems, seedling leaves. Messenger RNA levels were analyzed by semi-quantitative RT-PCR. The results show *OsBADH2 *is expressed constitutively, with less expression abundance in mature roots (Figure 1).

We examined genetic variation in a 463 bp portion of *OsBADH2 *gene in 13 fragrant and six non-fragrant rice accessions that are representatives of cultivated varieties across China (Table 1). Multiple mutations identical to *fgr *allele identified by Bradbury *et al*. [22] were also observed in all the 13 fragrant accessions (data not shown). These mutations contain a total of six SNPs and eight deletions within a 25 bp region of the exon 7 as compared to the wild type allele in the tested non-fragrant varieties.

**Table 1:** Varieties from across China used for determination of allelic variation of *OsBADH2 *locus.

**Accessions**	**Species**	**Properties**
Yuanlixiangjing	*Oryza sativa subs. japonica*	Aromatic
Yixiang 1	*Oryza sativa subs. indica*	Aromatic
Chuanxiang 29B	*Oryza sativa subs. indica*	Aromatic
Ganxiangnuo	*Oryza sativa subs. indica*	Aromatic, glutinous
Wanlixiang	*Oryza sativa subs. indica*	Aromatic
Zhongxiang 1	*Oryza sativa subs. indica*	Aromatic
Xinxiangzhan 1	*Oryza sativa subs. indica*	Aromatic
Zaoxiang 17	*Oryza sativa subs. indica*	Aromatic
Qimiaoxiang	*Oryza sativa subs. indica*	Aromatic
Nongxiang 16	*Oryza sativa subs. indica*	Aromatic
Neixiang 2A	*Oryza sativa subs. indica*	Aromatic
Neixiang 7A	*Oryza sativa subs. indica*	Aromatic
Yuxiangyouzhan	*Oryza sativa subs. indica*	Aromatic
Zhonghua 9	*Oryza sativa subs. japonica*	Non-aromatic
Yuelianggu	*Oryza sativa subs. japonica*	Non-aromatic
hongjiaomaojing	*Oryza sativa subs. japonica*	Non-aromatic
93-11	*Oryza sativa subs. indica*	Non-aromatic
Minghui 63	*Oryza sativa subs. indica*	Non-aromatic
Suhui 527	*Oryza sativa subs. indica*	Non-aromatic

### Deficient in OsBADH2 expression resulting in apparently sensory aroma production

**Figure 2:**
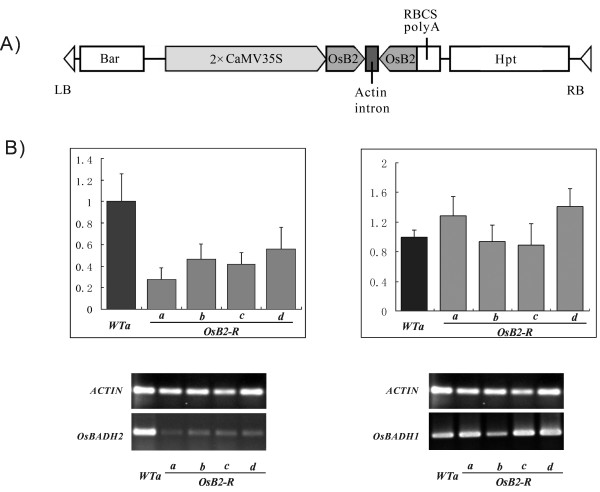
**Construct and molecular analysis of normal and transgenic plants. ***R *represents the presence of RNAi construct. Schematic diagram of part of the T-DNA region of the transforming construct *CMV35S-OsBADH2-RNAi *is shown in (A). Inversely repeated fragments derived from 3' coding region of *OsBADH2 *are indicated by *OsB2*. (B) Semi-quantitative RT-PCR, Real-time quantitative RT-PCR analysis of *OsBADH2*(left) and *OsBADH1*(right) mRNA levels in fully expanded leaves derived from wild-type (*WTa*) and four *OsBADH2*-deficient lines (*OsB2-Ra*, *b*, *c *and *d*). Each bar represents three replications from each RNA sample. Error bars represent standard errors shown in each case.

To dissect the physiological role of *OsBADH2*, we generated a large number of transgenic rice plants expressing *OsBADH2-RNAi *(Figure 2A). Construct was introduced into non-fragrant rice cv. Nipponbare by *Agrobacterium tumefaciens*-mediated T-DNA transfer. The majority of *OsBADH2-RNAi *primary transgenics (T0) resulted in PCR amplification with primers designed to the *hpt *(hygromycin phosphotransferase gene) marker. Preliminary aroma evaluation revealed that 21 out of 97 *hpt*-positive T0 plants showed abundant fragrance emission.

Ten T1 segregating populations derived from independent T0 plants with obvious aroma production were selected and cultivated in the university's farm. Leaves from the first and second crops and grains from the first crop of individual T1 plants were used for sensory evaluation of aroma production. As a result, the presence and absence of fragrance is unambiguously segregated within T1 populations and well consistently expressed in leaves and grains from the same plants. In all the 10 T1-segregating populations, we observed a correlation of *OsBADH2-RNAi *transgene integration with apparently sensory aroma production.

**Figure 3:**
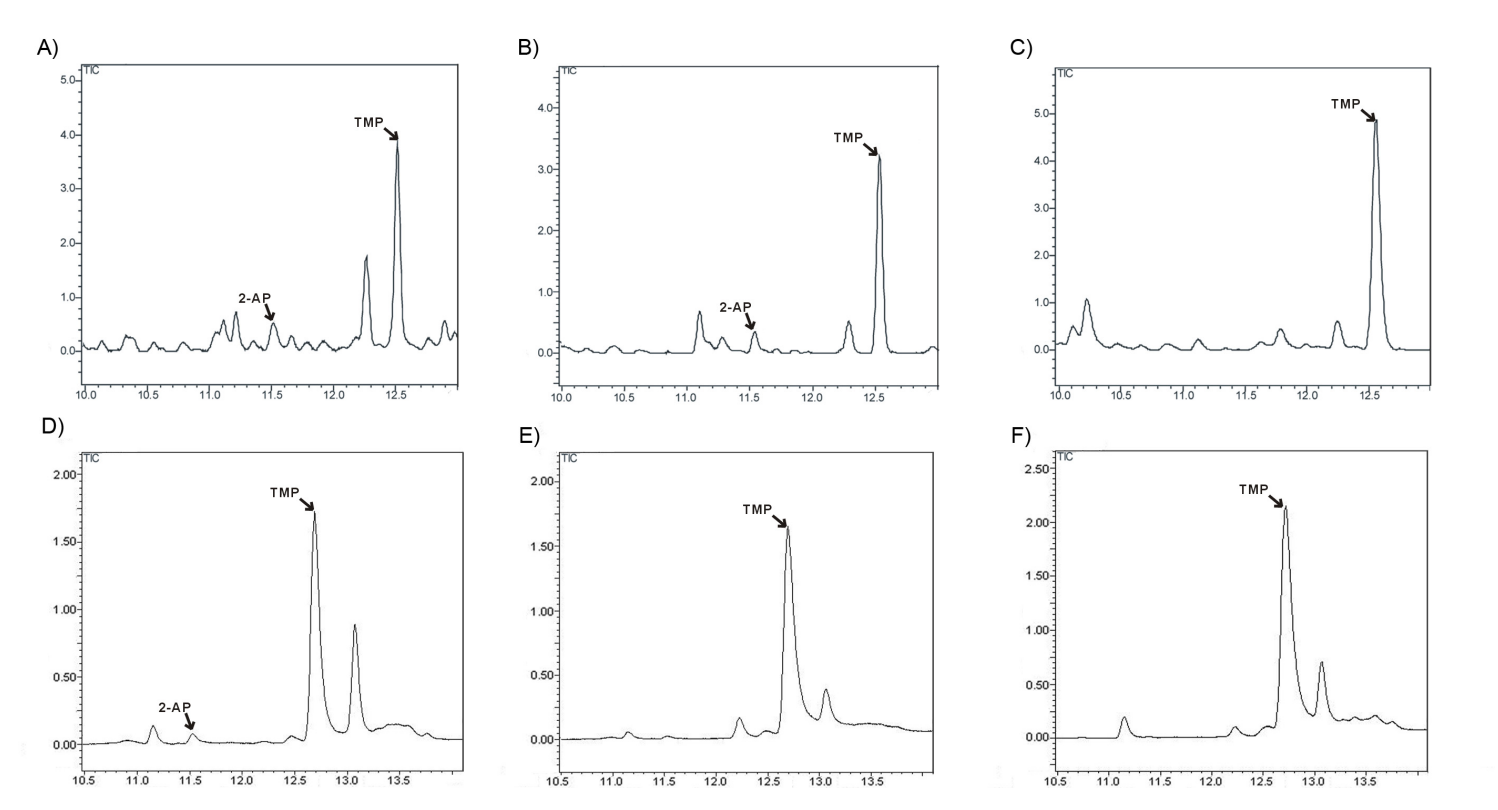
**Total ion chromatograms of extract solvent derived from Thai Hom Mali 105 (A), transgenic (B) and wild-type control cv.** Nipponbare (C) ground rice. Total ion chromatograms of extract solvent derived from fragrant (D), non-fragrant (E) transgenic progeny (*OsB2-Rc*) and wild-type control cv. Nipponbare (F) mature leaves. Marked peaks are indicated as 2-acetyl-1-pyrroline (2AP) and the 2, 4, 6-trimethylpyridine (TMP), respectively.

To investigate property of the emitted aromatic compound from the transgenic lines, a gas chromatography-mass spectrometry (GC/MS) analysis was conducted for Thai Hom Mali 105 (aromatic rice from Tailand), transgenic line (*OsB2-Rc*) and the wild type control. As shown in Figure 3, a considerable aroma production of 2-acetyl-1-pyrroline (2AP) was detected in both Thai Hom Mali 105 and transgenic line (*OsB2-Rc*), by contrast to the undetectable wild type control.

In order to test if the aroma production is co-segregated with the presence of T-DNA insertion in the transgenic segregating progeny, T2 mature leaves from five *OsBADH2-RNAi *repression plants and four non-fragrant wild-type plants segregated out from the same transgenic line (*OsB2-Rc*) were collected and subjected to the GC-MS analysis. As a result, 2AP accumulation was apparently enhanced in all the downregulation plants, by contrast to the undetectable wild-type plants without fragrance (Figure 3D–F).

To ensure that the observed phenotypes are correlated with reduced endogenous transcript, total RNA was extracted from leaves of wild type plants and four independent *OsBADH2-RNAi *repression lines. Analysis of semi-quantitative RT-PCR and real-time quantitative RT-PCR revealed a considerable reduction in endogenous *OsBADH2 *transcript levels in *OsBADH2-RNAi *repression lines compared to that of wild-type plants (Figure 2B).

To determine if the expression of *OsBADH1 *gene is affected in the down-regulated *OsBADH2 *transgenic lines, we conducted real-time PCR analysis to detect the *OsBADH1 *cDNA levels in the *OsBADH2-RNAi *repression lines. The *OsBADH1 *gene is the closest available homolog of *OsBADH2 *and is almost unaffected in the down-regulated *OsBADH2 *lines (Figure 2B).

### Disrupted OsBADH2 influencing crop productivity

To investigate the effects of down-regulated *OsBADH2 *on crop productivity, the plant height and 1000-grain weight of *OsBADH2*-deficient and wild type plants derived from four independent T2 transgenic lines were measured. The results showed the reduction in plant height as well as 1000-grain weight in the *OsBADH2*-deficient plants is differentially detectable when compared to the wild-type plants segregated out from the transgenic progenies (Table 2).

**Table 2:** Plant height and 1000-grain weight in segregated transgenic progenies

**Line**	**Plant height(cm) (n = 21)**		**1000-grain weight(g) (n = 21)**	
	**fragrant**	**non-fragrant**	**fragrant**	**non-fragrant**
*OsB2-Ra*	90.59 ± 4.39 *	98.30 ± 3.68	22.31 ± 1.27 *	23.51 ± 1.57

*OsB2-Rb*	96.12 ± 2.80 *	101.58 ± 3.07	22.92 ± 1.68 *	24.23 ± 1.57

*OsB2-Rc*	99.14 ± 2.76	100.84 ± 2.67	22.11 ± 1.15 *	24.01 ± 1.59

*OsB2-Rd*	94.78 ± 2.96	95.34 ± 4.66	20.91 ± 0.96 *	23.20 ± 1.59

* significant at P < 0.05 compared to non-fragrant wild-type plants segregated out from the same transgenic line.

**Figure 4:**
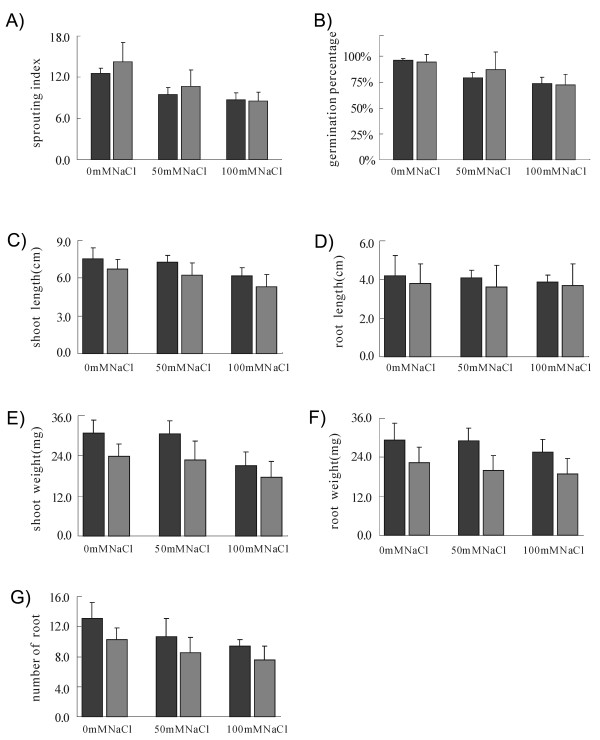
**Germination and seedling phenotypes of normal and transgenic plants.** Average performances were calculated for wild-type (*WTa*, *b *and *c*, black bars) and four *OsBADH2*-deficient lines (*OsB2-Ra*, *b*, *c *and *d*, grey bars), respectively. Comparison of sprouting index (A) and germination rates (B) between wide type control and transgenic lines is shown. Differences in seedling growth rates indicated as the length of shoot (C), length of root (D), fresh weight of shoot (E) and root (F), and the number of roots (G) are shown between wide type control and transgenics.

To further address the physiological functions of the *OsBADH2 *gene, wild type seeds and T2 seeds derived from four independent transgenic lines were germinated in various salt-stressed conditions. No significant difference in sprouting index and germination rates between wild type and *OsBADH2 *repression lines was observed (Figure 4A, B). Nevertheless, the seedling growth rates (scored by shoot length and weight, root length and weight and root number) in different salt concentrations were more or less inhibited in *OsBADH2*-deficient lines as compared to wild type (Figure 4C–G).

## Discussion

By using RNA interference (RNAi) technique in this experiment we have generated a number of transgenic plants expressing *CaMV35S-OsBADH2-RNAi *construct. Molecular analyses combined with panel sensory evaluation and gas chromatography-mass spectrometry demonstrates downregulation of *OsBADH2 *transcripts in the transgenic plants results in significant elevation of 2-acetyl-1-pyrroline production. This result is well consistent with the previous fine mapping data [21,22]. Interestingly, extensive sequence analysis in this and previous studies indicates that both the traditional and modern fragrant rice varieties with diverse origins possess the same mutant allele, suggesting the donor mutation leading to fragrance probably has a single evolutionary origin [22]. Distinctly, this spontaneous mutant allele prevalently present in all the tested fragrant rice varieties probably represents the capacity for plants to evolve phenotypic modifications in response to local cultural preferences. The mutant may occur even before rice domestication and disperse worldwide over the course of the domestication.

Rice *BADH2 *(*OsBADH2*) belongs to aldehyde dehydrogenases (ALDH) superfamily comprises a group of divergently related enzymes that catalyze the irreversible NAD(P)^+^-dependent oxidation of a wide variety of aliphatic and aromatic aldehydes to their corresponding carboxylic acids and occur in most well-studied organisms [23,24]. A distinct function of this gene family is involved in environmental stresses responses and tolerance [25,26]. Indeed, there are two closely related *betaine aldehyde dehydrogenase *(*BADH*) homologs (*OsBADH1 *and *OsBADH2*: accession nos. AK103582 and AK071221, respectively) present in rice genome [27]. Several studies showed that glycine betaine accumulation in rice (*O. sativa*) plants was undetectable, indicating a possible functional defect resulting from an unusual post-transcriptional processing at *choline monooxygenase *(*OsCMO*) and/or *betaine aldehyde dehydrogenase (OsBADH)* loci specifically for glycine betaine biosynthesis [28-31]. The current transgenic experiment demonstrates down-regulated *OsBADH2 *expression leads to reduced crop productivity, indicating a *bona fide *activity of the gene product encoded by *OsBADH2 *functioning in crop performance. Actually, the aldehyde dehydrogenase activity of OsBADH2 has been recently demonstrated in an independent study [32].

Intriguingly, a unique function of rice *BADH2 *involved in aroma production has been apparently implicated by RNAi-mediated expression repression. This result has been recently confirmed in an independent experiment [32]. At present, the biochemical pathway leading to aroma production in rice has not been established. The recessive nature of mutant fragrant allele suggests that a loss of function is responsible for the accumulation of fragrant compound [10-15,17]. In present experiment, down regulation of *OsBADH2 *mRNA level resulting in elevated aroma production further suggests the multiple mutations in the fragrant allele most likely give rise to a loss-of-function version of *OsBADH2 *and thereafter account for the aroma production. Accordingly, we are able to speculate the functional OsBADH2 protein encoded by the wild allele may catalyze a reaction that consumes either 2-acetyl-1-pyrroline or an upstream precursor of 2-acetyl-1-pyrroline in a competing pathway. Obviously, to further elucidate the biochemical pathway of 2-acetyl-1-pyrroline accumulation, additional gene loci involved in aroma metabolism need to be identified.

To harness the positive effects of *OsBADH2 *gene suppression in aromatic rice production without the collateral negative effects on crop performance, we could inhibit accumulation of the functional *OsBADH2 *mRNA either by RNA interference (RNAi) specifically in rice grain using *OsBADH2*-derived inverted-repeat constructs driven by endosperms-specific promoters, or by incorporating the loss-of-function spontaneous mutant aromatic allele into one of the parental lines in hybrid rice. Due to the recessive nature of spontaneous mutant aromatic allele, the vegetative growth of the heterozygous F1 plants of the hybrid rice varieties will not be adversely affected, while the endosperm homozygous for the mutant allele derived from the heterozygous F1 hybrids will produce and accumulate fragrant compound.

## Conclusion

This study indicates that down-regulated expression level of *OsBADH2 *gene favorably enhances the accumulation of aromatic compound, 2-acetyl-1-pyrroline. In addition, the sequencing data suggests the prevalent aromatic allele of spontaneous mutation has a single evolutionary origin.

## Methods

### Plant Materials and Growth Conditions

Thirteen fragrant and six non-fragrant cultivars from across China were used to determine the allelic variation of *OsBADH2 *locus. The plants were grown in farm's field at the Sichuan University. Primary transformants (T0) were first planted in the artificial climate incubators (BINDER, Tuttlington, Germany) under standard conditions (28°C day, 20°C night; 12 h light, 12 h dark), and transplanted into the field 5 weeks later. Wild type and the transgenic progeny plants were grown side by side in farm's field at the Sichuan University. After harvesting, the T1 stubs were transferred into pots and grown in a growth chamber (25°C, 12/12 h photoperiod at 200 μmol photons m^-2 ^s^-1^). Tillers were regenerated from the stubs. To investigate the germination and seedling phenotypes, wild type and T2 seeds derived from the T1 transgenic plants were germinated under various concentrations of NaCl in the growth chamber.

### Plasmid Construction and Rice Transformation

DNA manipulations were carried out by using standard procedures (Molecular Cloning). Sequences from *OsBADH2 *cDNA (GenBank accession no. AK071221) were amplified by RT-PCR for construction of *CaMV35S-OsBADH2-RNAi *vector. An inverted-repeat fragment was constructed into vector pSK int [33] and transferred into pHB (driven by 2 × CaMV35S promoter [34]) at the *Bam*H I and *Xba *I restriction sites by using PCR with primers (B2IF1: 5' GCTCTCGAGTCTAGACCAATGGCCAGATTTGCAGT 3'; B2IR1: 5' GCACAAGCTTTGCGAGCAGTTCACCCAGAT 3'; B2IF2: 5' GGTGGATCCCCAA TGGCCAGATTTGCAGT 3'; B2IR2: 5' CACGAATTCTGCGAGCAGTTCACCCAGAT 3') introducing unique restriction sites at the product ends.

Transgenic plants were generated by *Agrobacterium tumefaciens*-mediated transformation according to the described procedure [35], and transformed lines were first selected for hygromycin (50 mg L^-1^) resistance and then analyzed by PCR to determine the presence of T-DNA integration. Primers designed to the hygromycin phosphotransferase gene (*hpt*) of pHB for confirmation of integration are 5' TAGGAGGGCGTGGATATGTC 3' and 5' TACACAGCCATCGGTCCAGA 3' (GenBank accession No.E00777). PCR was performed by using Taq DNA Polymerase (Takara, Dalian, China) in MJ Mini™PCR (BIO-RAD, Hercules, California, USA), following the instruction given by the manufacturer.

### Molecular analyses

Genomic DNA was extracted from mature rice leaves to determine the allelic variation of *OsBADH2 *locus in different varieties (Table 1). A 463 bp portion of *OsBADH2 *gene was amplified by PCR with primers designed for the *OsBADH2 *(5' ACATAGTGACTGGATTAGGTTCTG 3' and 5' CATCAACATCATCAAACACCACT 3'). This region of the gene includes the previously identified mutations putatively responsible for the aroma production [22]. PCR products were cloned into the pMD18-T Vector (Takara, Dalian, China). Cycle sequencing was performed with the ABI Prism BigDye Terminators v2.0 cycle sequencing reaction kit (Applied Biosystems, Foster City, California, USA). Sequences were determined with an ABI Prism 377 genetic analyzer (Applied Biosystems). The sequence analysis was performed using the software DNAMEN v5.0 (Lynnon Biosoft Inc., Vandreuil, Quebec, Canada).

For semi-quantitative RT-PCR, total RNAs were extracted using Trizol reagent following the protocol provided by the manufacturer (Invitrogen, Carlsbad, CA) and treated with DNase (TaKaRa, Dalian, China). About 1 μg of total RNA from each sample was used for first-strand cDNA synthesis (Toyobo, Osaka, Japan). *ACTIN *gene was employed as positive internal control [36]. Primers used for the RT-PCR analysis were designed for the *ACTIN *(5' AAGATCCTGACGGAGCGTGGTTAC 3' and 5' CTTCCTAATATCCACGTCGCACTTC 3'), and *OsBADH2 *(5' CCAATGGCCAGATTTGCAGT 3' and 5' TGCGAGCAGTTCACCCAGAT 3'), *OsBADH1 *(5' TGCGAACGCTGGTCAAGTCT 3'; 5' ATCACAGCGCCAGCTAGACC 3'). For real-time quantitative RT-PCR, total RNAs were treated with DNase (TaKaRa, Dalian, China) and about 1 μg of total RNA from each sample was used for first-strand cDNA synthesis (Toyobo, Osaka, Japan). Primers for real-time quantitative RT-PCR were designed for *OsBADH2 *(5' TGAAGCCGGTGCTCCTTTGT 3'; 5' CACTATAGGACTTTTTCCACCAAG 3'), *OsBADH1 *(5' GGTCAAGCCTGTTTCGTTAGAG 3'; 5' CAACCAACCTATCCAAGAATCGCT 3'), and the control *ACTIN *(5' ACCTTCAACACCCCTGCTAT 3'; 5' CACCATCACCAGAGTCCAAC 3'). The real time quantitative PCR was carried out in a total volume of 25 μL containing 12.5μL of SYBR^® ^Premix Ex Taq TM (TaKaRa, Dalian, China), 0.2 μM of each primer, and 7.5 μL of 1:65 diluted cDNA. Thermal cycling consisted of a hold at 95°C for 40 seconds followed by 40 cycles of 95°C for 5 seconds, 57°C for 10 seconds and 72°C for 15 seconds. After amplification, samples were kept at 95°C for 1 min and 55°C for 1 min. Then the temperature was raised gradually by 0.5°C every 10 seconds to perform the melt-curve analysis. Each sample was amplified in triplicate and all PCR reactions were performed on the iCycler^®^PCR system (BIO-RAD, Hercules, California, USA). REST software [37] was used to quantify the *OsBADH2 *and *OsBADH1 *mRNA levels with *ACTIN *normalization by the 2-Ct method. To confirm the specificity of the PCR reaction, PCR products were electrophoresed on 1% agarose gel to verify accurate amplification product size.

### Sensory aroma evaluation

Determination for the presence or absence of aroma was made according to previously described methods [38]. Basmati370 was used as a control for the sensory aroma evaluation. At tillering stage of transgenic plants, 2 g fresh leaves were excised from individual plants, cut into 5 mm long pieces and kept in petri dishes mixed with 10 ml of 1.7% potassium hydroxide (KOH) solution. The petri dishes were kept under room temperature for about 10 minutes. They were then opened one by one, and the samples were sniffed and scored for presence or absence of aroma emission. To confirm the presence or absence of aroma, leaf tissue from tillers regenerated from stubs of T1 plants were soaked in KOH solution and evaluated for the aroma emission. In addition, about 20 grains harvested from T1 plants were placed into 5-ml screw-cap tube containing 1 ml of fresh water and were then incubated at 65°C for 2 hours. The aroma was evaluated after storage at 4°C for 20 min [21]. All the samples were sniffed by three well-trained panelists. The samples were classified into two categories in presence or absence of aroma.

### Gas chromatography-mass spectrometry (GC-MS)

The hulled rice grains and/or mature leaves from the *OsBADH2-RNAi *transgenic and wild-type *japonica *cv. Nipponbare were collected, and Thai Hom Mali 105, a commercial aromatic variety from Thailand purchased from a retail store, was used as control [39]. The 2, 4, 6-trimethylpyridine (TMP, Sigma Aldrich Chemical Co., Germany) was used as an internal standard [39,40]. It was dissolved in a precisely measured volume of 0.1 M HCl to give an internal standard solution with 2.00 ppm concentration of TMP. The milled rice grains/leaves (30 g) were added to a 250 mL flask containing 120 mL of internal standard solution. The mixture was stirred for 2 h before filtration. 9 mL of 1.0 M NaOH was added to the filtrate to make the solution slightly basic, then transferred to two 50 mL centrifuge tubes and centrifugated at 8000 rpm for 10 min. About 80 mL of the supernatant liquor was transferred to a 250 mL pear-shaped separatory funnel. Then 120 mL of dichloromethane was immediately added as an organic solvent. The extraction was conducted twice, resulting in 240 mL of dichloromethane solution. After drying with anhydrous sodium sulfate, the extract was concentrated to 1 mL using a rotary evaporator under reduced pressure and a temperature of 26°C. The concentrated extract was transferred to a tube and left open to the air at room temperature until its volume decreased to 0.1 mL, from which 1 μL was taken for qualitative analysis.

Samples were determined on a GC/MS system (GC-MSQP2010W, Shimadzu, Japan). Helium gas (purity 99.999%) at a pressure of 80 Kpa was used as the GC carrier gas. The injector and the GC/MS interface temperatures were set at 170°C and 250°C, respectively. The temperature of Rtx-wax capillary colum (30 m × 0.25 mm id, film thickness 0.25 μm, Restek, Bellefonte, PA) was programmed starting at 40°C after injection of samples. With the initial temperature of 40°C held for 2 min, it was ramped to 80°C at 6°C/min. After hold for 1 min in 80°C, it increased to 120°C at a rate of 4°C/min, then mounted up to 200°C at 8°C/min and held there for 20 min. The effluent from the capillary column went directly into the masspectrometer, operated in the electron impact (EI) mode with an ionization voltage of 70 eV, and the ion source temperature was 200°C [39,40].

### Agronomical trait measurements

To investigate the effects of the disrupted *OsBADH2 *on crop performance, agronomical traits including plant height and 1000-grain weight were measured by using T2 fragrant/non-fragrant segregated progeny plants derived from four independent primary transformants.

### Evaluation of germination and seedling growth rates

To compare grain sprouting rates between wide type and *OsBADH2-RNAi *repression lines, 50 grains from each of three wild type controls (*WTa*, *b *and *c*) and four *OsBADH2*-deficient lines (*OsB2-Ra*, *b*, *c *and *d*) were placed and incubated in Petri dishes containing two pieces of filter paper moistened with 0, 50, 100 mM NaCl for 8 days. Sprouting index (∑R_GT_/t, R_GT_, the number of spouted grains in the t day) and germination rates (G/N × 100%, G, the number of spouted grains; N, the total number of grains) were measured.

For seedling growth rate experiments, 30 grains from each of three wild type controls (*WTa*, *b *and *c*) and four *OsBADH2*-deficient lines (*OsB2-Ra*, *b*, *c *and *d*) were incubated in distilled water for 7 days, and subsequently subjected to different salt concentrations (0, 50, 100 mM NaCl) for another 7 days. The length of shoots and roots, the number of roots and the fresh weight of shoots and roots were examined, respectively.

## Authors' contributions

YL and TL conceived the study, designed the experiments and drafted the manuscript. XN, WT and WH conducted the plasmid construction, transformation, RT-PCR and real-time PCR analyses, phenotypic assay and GC-MS analysis. BRL, GR participated in designing the experiments. FW and QW participated in transformation. DL, YX, SY, and FG carried out the aroma evaluation, crop performance analysis and field data collections.
